# Transforming language assessment in the digital age: university-level EFL students' and teachers' perceptions of traditional, alternative, and online assessment models

**DOI:** 10.3389/fpsyg.2026.1851149

**Published:** 2026-05-22

**Authors:** Omid Nassery, Necdet Osam

**Affiliations:** Department of Foreign Language Education, Faculty of Education, Eastern Mediterranean University, Gazimagusa/Northern Cyprus, Mersin, Türkiye

**Keywords:** English as a Foreign Language, fairness, language assessment, motivation, technology-mediated learning

## Abstract

**Introduction:**

English-Medium Instruction (EMI) contexts in multicultural higher education settings present unique challenges for language assessment, particularly regarding student motivation, perceived fairness, and effort allocation. Despite growing adoption of technology-mediated assessment models, systematic comparisons of traditional, alternative, and online assessment approaches from both student and instructor perspectives remain limited. Grounded in the Technology Acceptance Model (TAM), this study examines how technology integration transforms language assessment processes in university-level EFL (English as a Foreign Language) education. This study aimed to (a) compare undergraduate students' and faculty members' perceptions of traditional, alternative, and online assessment models across three dimensions—motivation, perceived fairness, and required effort; (b) examine intergroup differences between students and instructors; and (c) investigate associations among motivation, fairness, and effort perceptions to inform pedagogical recommendations for hybrid assessment design in EMI contexts.

**Methods:**

A cross-sectional survey design was employed with 258 undergraduate students and 32 faculty members at Eastern Mediterranean University (Northern Cyprus), all engaged in English-medium instruction. Participants completed a validated quantitative instrument measuring perceptions of the three assessment models across motivation, fairness, and effort dimensions. Descriptive statistics, independent-samples t-tests (or Mann-Whitney U tests), and Pearson correlation analyses were conducted using SPSS 30.0. The study was conducted in accordance with ethical guidelines, with informed consent obtained from all participants.

**Results:**

Students perceive all three assessment models as moderately motivating, whereas teachers are more skeptical of the motivational effectiveness of alternative assessments. While both groups rated alternative assessments as the fairest model, students have lower perceptions of the fairness level of online assessments. Significant differences were observed in perceptions of effort: students perceive traditional assessments as requiring the most effort, whereas teachers perceive alternative assessments as requiring more effort. Correlation analyses reveal strong positive associations between motivation and effort (*r* = .49-.50), and moderate associations between fairness and motivation (*r* = .39-.41). The study highlights the differences between student and teacher perceptions in the context of technology-mediated language assessment and provides pedagogical recommendations for the design of hybrid and flexible assessment strategies.

**Discussion:**

The findings demonstrate the critical role of perceived fairness in motivation and learning outcomes, particularly in multicultural English-Medium Instruction (EMI) contexts. The research is discussed in comparison with current literature on how technology integration is transforming language assessment processes, and future research directions and application recommendations are presented.

## Introduction

Language assessment constitutes a fundamental mechanism through which educational institutions evaluate learner progress, certify achievement, and shape instructional priorities. In English as a Foreign Language (EFL) contexts, assessment serves a dual function: measuring linguistic competence and motivating sustained engagement with the target language (Bachman and Palmer, 2010; Black and Wiliam, 1998). Contemporary language assessment has diversified into three predominant models: traditional assessments (standardized tests, multiple-choice examinations), alternative assessments (portfolios, presentations, project-based evaluations), and online assessments (digital tests, e-portfolios, automated evaluation systems). Each model embodies distinct epistemological assumptions regarding the nature of language proficiency, the role of the learner, and the locus of evaluative authority (Scarino, 2013; Phongskirikul, 2018).

The COVID-19 pandemic precipitated an unprecedented acceleration in the adoption of digital assessment platforms, transforming what had been an emergent pedagogical option into an institutional necessity (Ng et al., 2021; Alenezi, 2022). In the post-pandemic era, online and hybrid assessment models have become permanently integrated into education systems, rendering the concept of technology-mediated language assessment central to both research and practice (Gavrilau et al., 2025). However, this technological transformation has not occurred without friction. Students from diverse cultural backgrounds frequently perceive online assessments as less fair than traditional in-person evaluations, raising questions of equity and inclusivity that extend beyond technical infrastructure to encompass social and cultural dimensions (Joo et al., 2024; Jia and Hew, 2022).

Despite the growing body of research on individual assessment modalities, systematic comparative studies examining stakeholder perceptions across all three models—traditional, alternative, and online—within a single multilingual, English-Medium Instruction (EMI)-focused context remain scarce. Existing research has tended to examine these models in isolation or to focus on a single stakeholder group, obscuring the interactional dynamics between student expectations and teacher beliefs that ultimately determine assessment effectiveness (Guo, 2025; Xu and Brown, 2016). Furthermore, the theoretical foundations underlying assessment perception research remain underdeveloped; while numerous studies describe what stakeholders prefer, few articulate the psychological mechanisms through which assessment format shapes motivation, fairness perception, and effort allocation.

This study addresses these gaps by examining how university-level EFL students and instructors at Eastern Mediterranean University (EMU), a multicultural English-Medium Instruction institution in Northern Cyprus, perceive traditional, alternative, and online assessment models across three dimensions: motivation, fairness, and effort. Grounded in the Technology Acceptance Model (TAM) (Davis, 1989; Davis and Venkatesh, 2004), the study investigates how perceived usefulness and perceived ease of use—adapted here to the assessment context—mediate stakeholder acceptance of different evaluation formats. Theoretically, the study extends TAM into the domain of language assessment by demonstrating that perceived fairness functions as a critical antecedent to both perceived usefulness and behavioral intention to engage. Methodologically, it employs a cross-sectional questionnaire design with a culturally heterogeneous sample, offering greater ecological validity than studies conducted in monocultural contexts. Practically, the findings inform the design of hybrid assessment strategies that balance accountability, learner autonomy, and technological accessibility in EMI environments. By revealing systematic divergences between student and teacher perceptions, the study contributes to the development of more inclusive, responsive, and pedagogically sound assessment ecosystems.

## Literature review and theoretical framework

### Language assessment models: from traditional to digital

Assessment in language education has traditionally been associated with standardized, summary formats such as multiple-choice tests, true/false items, closure tasks, and timed essays; these formats are designed to measure learners' proficiency at a fixed point in time (Irawan, 2017; Brown, 2004). These traditional assessments are often praised for their reliability and easy implementation, especially in large-scale testing environments. However, they have been consistently criticized for their limited construct validity and inability to capture communicative competence or authentic language use (Rea-Dickins and Gardner, 2000; Shohamy, 2001).

In contrast, alternative, or performance-based, assessments evaluate language skills in real-world or process-oriented contexts. These may include portfolios, peer and self-assessments, oral presentations, and project-based work (Cheng et al., 2004; Phongskirikul, 2018). Proponents argue that alternative assessments promote student autonomy, critical thinking, and meaningful feedback (Alenezi, 2022; Hamp-Lyons, 2007). Despite this, it is often considered time-consuming and poses challenges to standardization, consistency, and teacher assessment literacy (Looney et al., 2018).

Global change accelerated by the COVID-19 pandemic has further diversified the areas of assessment. Online assessments can mimic traditional formats digitally or incorporate alternative methods, such as e-portfolios, discussion boards, and online collaboration tasks (Ng et al., 2021; Khalil and Ebner, 2020). While these platforms offer enhanced accessibility and flexibility, they also raise new concerns about academic integrity, technological access, and student digital competence (Alenezi, 2022; Alderson and Banerjee, 2001).

In the post-pandemic era, research on online assessment has gained significant momentum. Ng et al. (2021) conducted a comprehensive comparison of traditional in-person and online assessments, revealing that online environments are perceived as less fair by students but offer greater accessibility and flexibility. Gavrilau et al. (2025), on the other hand, conducted a meta-analysis of the effectiveness of online assessment in language learning, noting that although learners generally viewed online assessments positively, this view varied by assessment type and application context. These findings suggest that online assessment is not a universal solution but rather requires careful pedagogical planning.

### Evaluation perceptions of students and teachers

Students' perception of assessment significantly influences their motivation, engagement, and academic behavior (Carless, 2006). Research indicates that while students often acknowledge the objectivity and predictability of traditional assessments, they prefer alternative formats to promote deeper learning and more meaningful feedback (Phongskirikul, 2018; Cheng and Warren, 2005). Online assessments, initially faced with uncertainty, are increasingly accepted for their convenience—but concerns about fairness and technical reliability persist (Ng et al., 2021).

Teachers' perceptions are shaped by their assessment concepts and institutional experiences (Xu and Brown, 2016). Guo (2025) observed that university EFL instructors often adopt blended assessment philosophies that balance summative and formative goals. Traditional assessments are often regarded as efficient and scalable (Iqbal and Manarvi, 2011), whereas alternative assessments are often perceived as subjective and labor-intensive, despite their pedagogical merit (Cheng et al., 2004). Online assessment formats elicit mixed reactions—praised for automation and data management, while criticized for integrity risks and the need for new assessment competencies (Alenezi, 2022; Deneen and Boud, 2014).

Recent research highlights that teachers' digital assessment literacy is becoming increasingly critical. Yanik and Kiliç (2025) examined how AI-assisted assessment tools are perceived by teachers, revealing that they generally exhibit a positive yet cautious approach toward these technologies, with ethical concerns and concerns about loss of pedagogical control. Similarly, Jia and Hew (2022) showed that perceived benefit and perceived ease of use play decisive roles in teachers' adoption of online assessment tools.

### Artificial intelligence and automated writing evaluation systems

Over the past 5 years, AI-powered assessment systems have been driving revolutionary changes in language education. AWE systems provide instant feedback to students and have the potential to reduce teachers' workload (Li, 2025). However, Li et al. (2024) note that teachers have a dual-pronged attitude toward AWE systems—both recognizing their potential benefits while also worrying about restricting student creativity and reducing the quality of feedback.

Kessler (2024) has examined the implications of ChatGPT and similar large language models in language teaching and discussed how these technologies are transforming assessment practice. The research highlights that while AI-powered tools offer personalized learning experiences for students, they also raise fundamental questions such as academic integrity, plagiarism, and original thinking. Joo et al. (2024) examined the effectiveness of AI-driven feedback systems in multicultural contexts, revealing that cultural differences significantly influence the perceived utility and utilization of these technologies.

### Comparative analysis of assessment models

A comparative analysis of assessment types reveals both possibilities and constraints. Traditional assessments offer high reliability but may not adequately represent complex language performance. While alternative assessments encourage engagement and formative feedback, they raise questions of inter-rater reliability and teacher workload. Online assessments expand access and flexibility, but they are highly dependent on infrastructure, security protocols, and user readiness (Looney et al., 2018; Scarino, 2013).

Although many studies have examined individual assessment modalities, few have systematically compared student and teacher perceptions in a single multilingual, EMI-focused context across the three models. As Guo (2025) argues, contextualizing assessment beliefs is crucial to advancing pedagogically sound, culturally sensitive assessment practices. By examining how key stakeholders interpret and evaluate different assessment tools, researchers can ensure the development of hybrid assessment strategies that promote both accountability and learner-centricity.

This study bridges this gap by examining how university-level EFL students and instructors perceive traditional, alternative, and online assessments. The following section presents the TAM as the theoretical framework grounding this investigation, followed by an outline of the methodological approach to examine their attitudes toward motivation, fairness, and effort—the three integral dimensions of effective and inclusive evaluation.

### Theoretical foundation: technology acceptance model-TAM

TAM, originally developed by Davis (1989) and subsequently refined by Davis and Venkatesh (2004), provides the theoretical foundation for this study. TAM posits that individuals' acceptance and use of technology are determined by two primary beliefs: perceived usefulness (PU) and perceived ease of use (PEOU). Perceived usefulness refers to the degree to which a person believes that using a particular technology will enhance their job performance or learning outcomes. Perceived ease of use refers to the degree to which a person believes that using the technology will be effort-free. These beliefs jointly influence attitudes toward technology, which in turn shape behavioral intentions and actual usage behavior.

In the context of language assessment, TAM has been adapted to explain how students and teachers accept or resist various assessment technologies. Perceived usefulness in assessment corresponds to the belief that a particular assessment format validly measures language competence and contributes to learning improvement. Perceived ease of use corresponds to the cognitive and logistical demands associated with preparing for, administering, or completing the assessment. The present study extends TAM by introducing perceived fairness as a critical antecedent variable. Drawing on organizational justice theory (Colquitt et al., 2001), we argue that fairness perceptions—encompassing distributive justice (equity of outcomes), procedural justice (transparency of processes), and interactional justice (quality of interpersonal treatment)—function as a precondition for the formation of positive usefulness and ease-of-use beliefs. An assessment format perceived as unfair, regardless of its technical sophistication, will fail to generate the motivational engagement necessary for valid measurement.

This tripartite theoretical framework—integrating TAM's utility and usability dimensions with justice theory's fairness dimension—offers predictive and explanatory power that purely descriptive studies of assessment preference lack. It generates testable hypotheses: (1) assessment formats perceived as more useful and easier to use will be associated with higher motivation; (2) fairness perceptions will mediate the relationship between assessment format and motivation; and (3) the relative weight of usefulness, ease of use, and fairness will differ between students and teachers due to their divergent roles in the assessment process.

## Method

### Research design

This study adopted a cross-sectional questionnaire design to examine the attitudes of English learners and instructors toward the three main assessment models (Dörnyei, 2007). A structured questionnaire served as the primary data collection tool, designed to collect quantitative data using five-point Likert-scale items. These items measured participants' levels of agreement with statements about their assessment experiences and perceptions.

The instrument is focused on three key dimensions central to its assessment:

1. *Motivational value*—the degree to which assessment types encourage preparation and engagement.
2. *Perceived fairness*—participants' judgments about equity and transparency in the judging process.
3. *Effort required*—the amount of cognitive or physical preparation required for each assessment model.

The aim was to identify differences and commonalities in how students and teachers interpret these dimensions in traditional, alternative, and online formats. Likert-scale responses provided standardized measurement and comparability across participant groups (Cohen et al., 2018).

### Research context

To ensure ecological validity, the study was conducted at EMU, a leading EMI institution in Northern Cyprus. EMU was chosen for its diverse academic environment and extensive use of three assessment models in its English language programs. As a member institution of the Bologna Process, EMU implements formative and summative assessments across traditional, alternative, and online modalities.

A key criterion for institutional selection was the presence of teacher and student populations with demonstrable experience in multiple assessment formats. EMU's students and teaching staff from more than 100 nationalities have provided a linguistically and culturally heterogeneous sample, thereby increasing the study's generalizability.

### Participants

Participants were selected through a combination of convenience and purposive sampling strategies (Table 1). Eligibility criteria are required to ensure that all participants have relevant experience of traditional, alternative, and online assessments:

**Table 1:** Participant demographics.

Feature	Students (*n* = 258, %)	Teachers (*n* = 32, %)
Gender		
Female	142 (%55.0)	18 (%56.3)
Male	116 (%45.0)	14 (%43.7)
Academic year		
Year 1	68 (%26.4)	—
Year 2	89 (%34.5)	—
Year 3	62 (%24.0)	—
Year 4	39 (%15.1)	—
**Age range**	18–24 years (*M* = 20.3, *SD* = 1.7)	28–56 years (*M* = 41.2, *SD* = 8.4)
Teaching experience		
2-5 years	—	12 (%37.5)
6-10 years	—	14 (%43.8)
11+ years	—	6 (%18.7)
Main regions		
Middle east	178 (%69.0)	22 (%68.8)
Africa	52 (%20.2)	6 (%18.7)
Europe/Asia	28 (%10.8)	4 (%12.5)

Regional categories reflect participants' self-reported countries of origin. Due to relatively low frequencies, participants from Europe and Asia were combined into a single category (Europe/Asia) for descriptive reporting. The Middle East category includes participants from countries such as Türkiye, Iran, Syria, Jordan, Lebanon, and the Gulf states. The Africa category includes participants from Nigeria, Ghana, Cameroon, Sudan, and other sub-Saharan and North African countries.

- *For students*, at least one full academic year of English language study at EMU and previous experience with three assessment models.
- *For tutors*, a minimum of two years' teaching experience, including the systematic use of traditional, alternative, and online assessments in both formative and summative contexts.

Written informed consent was obtained from all participants prior to data collection. Students provided their own consent, as all participants were adults (aged 18 years or older) enrolled in university-level education. No legal guardians or next of kin were involved in the consent process.

The student sample was drawn from various academic departments, including Computing and Technology, Business Administration, Tourism, Pharmacy, Civil Engineering, Architecture, Electrical Engineering, English Language Teaching, and Psychology. These departments were selected because their curricula systematically incorporate all three assessment models across multiple courses. The teacher sample (*n* = 32) comprised full-time teachers of Foreign Languages and English at the EMU Foreign Languages and English Preparatory School who teach English in their respective departments. The age range for student participants was 18–24 years (*M* = 20.3, *SD* = 1.7), while teacher participants ranged from 28 to 56 years (*M* = 41.2, *SD* = 8.4).

The final sample consisted of 258 undergraduate students of predominantly Middle Eastern and African descent, with a smaller group of English faculty members (*n* = 32). The eligibility criterion of “at least one full academic year of English language study at EMU” was operationalized as completion of two consecutive academic semesters of English-medium coursework, including exposure to all three assessment models. Therefore, first-year students included in the sample were enrolled in the second semester of their first academic year at the time of data collection and had already experienced the required assessment formats (*n* = 68). All participants confirmed prior experience with traditional assessments (e.g., midterm/final examinations), alternative assessments (e.g., portfolios, presentations), and online assessments (e.g., digital quizzes, e-portfolios) through a screening question at the beginning of the questionnaire.

The instructors had at least 3 years of teaching experience at EMU and diverse cultural and linguistic backgrounds, reflecting the university's broader demographics.

### Data collection tools

The data collection tool consisted of a custom-designed questionnaire divided into two parts:

- *Part A:* Demographic information (e.g., gender, nationality, academic status, years of experience).
- *Part B:* Items on a 5-point Likert scale that assess participants' perceptions of traditional, alternative, and online assessments. Each evaluation model was assessed for motivation, fairness, and effort.

The final instrument comprised 45 Likert-scale items: 15 per assessment model (traditional, alternative, online) and 5 per dimension (motivation, fairness, effort). Example items include:

- **Motivation (Traditional):** “*traditional exams motivate me to study regularly throughout the semester.”*
- **Motivation (Alternative):** “*working on portfolios or projects makes me more interested in improving my English.”*
- **Motivation (Online):** “*taking online assessments encourages me to manage my own learning.”*
- **Fairness (Traditional):** “*traditional exams are graded fairly because all students answer the same questions.”*
- **Fairness (Alternative):** “*Alternative assessments are fair because they allow me to show my true language abilities.”*
- **Fairness (Online):** “*online assessments are fair because all students have equal access to the test platform.”*
- **Effort (Traditional):** “*preparing for traditional exams requires a lot of memorization and practice.”*
- **Effort (Alternative):** “*completing alternative assessments requires sustained effort over a long period.”*
- **Effort (Online):** “*online assessments require less physical effort because I can take them from anywhere.”*

The development of the instrument followed a multi-stage verification process:

1. *Expert review:* in the language assessment, four experts evaluated the questionnaire for the validity, clarity, and relevance of the content.
2. *Pilot testing:* a small sample of the target population (*n* = 30) completed the draft device, leading to minor revisions.
3. *Exploratory Factor Analysis (EFA):* conducted to assess construct validity and item alignment with the intended dimensions.
4. *Reliability analysis:* The latest version has provided strong internal consistency; Cronbach's alpha = 0.92 ratio indicates high reliability.

### Data analysis

The collected data were analyzed using SPSS 30.0. Descriptive statistics (mean, standard deviation, frequencies) were used to summarize participant characteristics and key trends. Independent sample *t*-tests and one-way ANOVAs were applied to identify differences between student and teacher groups. Pearson correlation coefficients were calculated to assess relationships among variables. The correlation matrix is presented as a heat map to visualize the intrinsic relationships between variables.

## Results

### The motivational role of assessment tools

**Figure 1:**
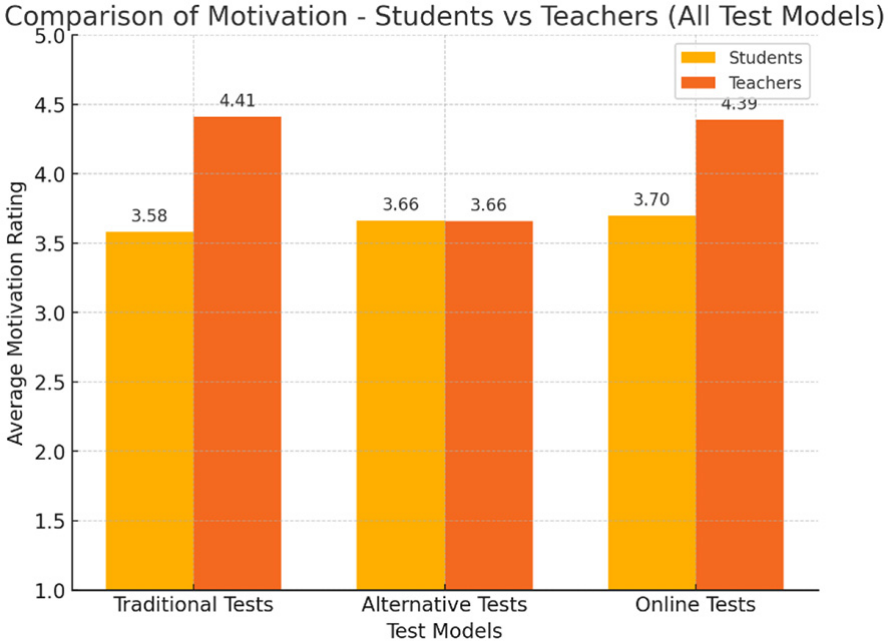
Comparison between students' and teachers' attitudes toward the motivating nature of test models. Teachers rated alternative assessments as less motivating than traditional and online formats (*M* = 2.94), whereas students perceived all three formats as similarly motivating (*M* = 3.42–3.45).

Responses to the Likert-scale items measuring perceptions of motivation regarding the exam showed differences between students and teachers (Figure 1). Both groups reported moderate differences in motivational impact across test types. However, teachers rated alternative assessments as less motivating than traditional and online formats; students perceived all three formats as equally motivating (Table 2).

**Table 2:** Perceptions of motivation, fairness, and effort for evaluation models (*mean* and standard deviation-*SD*).

Size	Evaluation model	Students	Teachers
		*Mean* (*SD*)	*Mean* (*SD*)
**Motivation**	Traditional	3.42 (0.78)	3.38 (0.82)
	Alternative	3.45 (0.81)	2.94 (0.89)
	Online	3.39 (0.85)	3.21 (0.76)
**Fairness**	Traditional	3.60 (0.72)	3.52 (0.68)
	Alternative	3.63 (0.69)	3.71 (0.74)
	Online	3.29 (0.88)	3.48 (0.71)
**Effort**	Traditional	3.60 (0.75)	3.22 (0.69)
	Alternative	3.15 (0.82)	3.91 (0.85)
	Online	3.28 (0.79)	2.89 (0.73)

All items are rated on a 5-point Likert scale (1, Strongly disagree; 5, Strongly agree).

### Perceptions of justice

**Figure 2:**
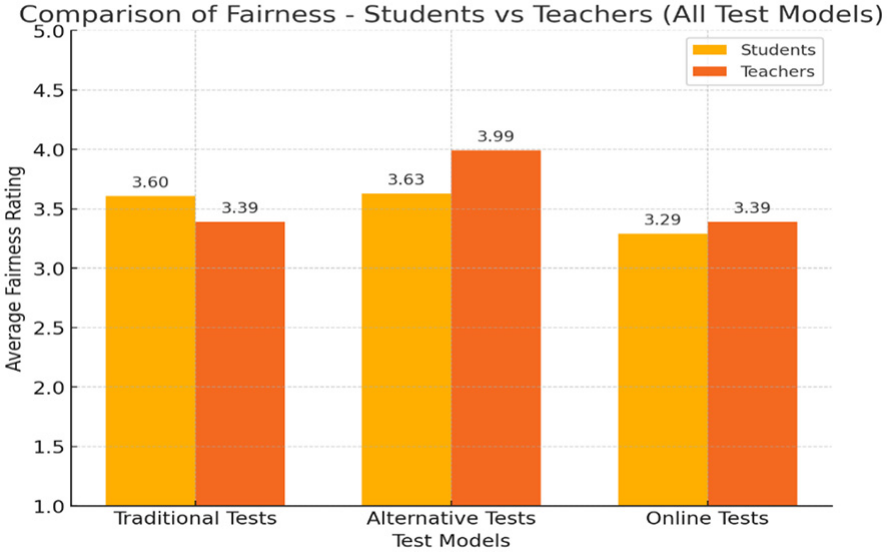
Attitudes of students and teachers toward the fairness of different test models. The figure shows that students perceive alternative assessments as the fairest (*M* = 3.63) and online assessments as the least fair (*M* = 3.29). Teachers rated alternative assessments as the fairest (*M* = 3.71), but found traditional (*M* = 3.52) and online (*M* = 3.48) formats equally fair.

Participants also rated each assessment model on perceived fairness. Students rated alternative assessments (*M* = 3.63) and traditional assessments (*M* = 3.60) as fairer than online assessments (*M* = 3.29). Teachers also supported alternative assessments as the most equitable, but rated traditional and online formats as equally fair (Figure 2).

### Perception of required effort

**Figure 3:**
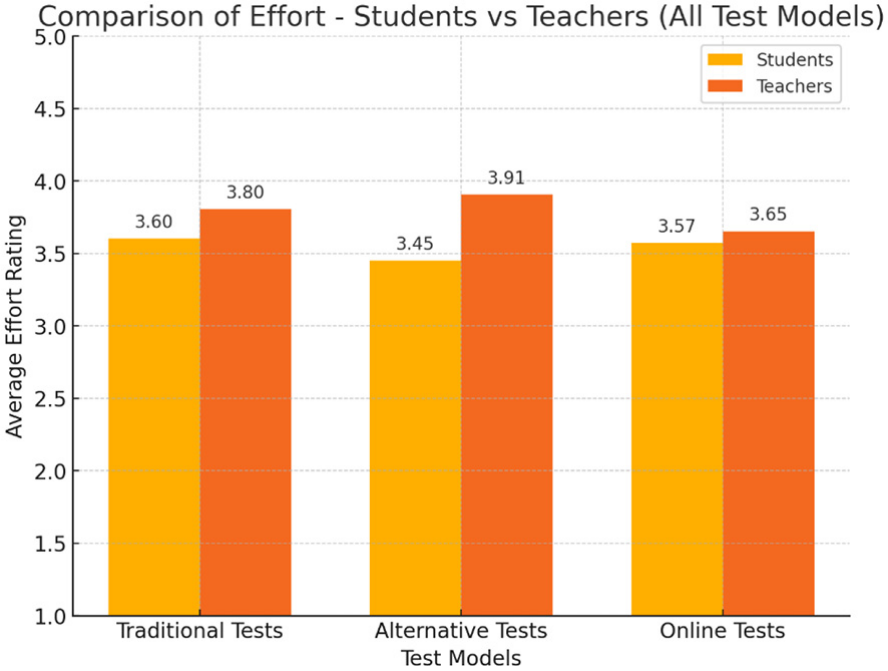
Students' vs. teachers' attitudes toward the effort required for each test model. The figure shows that students perceive traditional assessments as requiring the most effort (*M* = 3.60), while teachers rate alternative assessments as requiring the most effort (*M* = 3.91). Teachers perceived online assessments as requiring the least effort (*M* = 2.89).

Participants' perceptions of the effort required to complete each type of assessment varied significantly. Students rated traditional assessments as requiring the most effort (*M* = 3.60), followed by online and alternative formats. In contrast, teachers rated alternative assessments as requiring the most effort (*M* = 3.91), followed by traditional and online tests (Figure 3).

### Correlational analysis of evaluation perceptions

**Figure 4:**
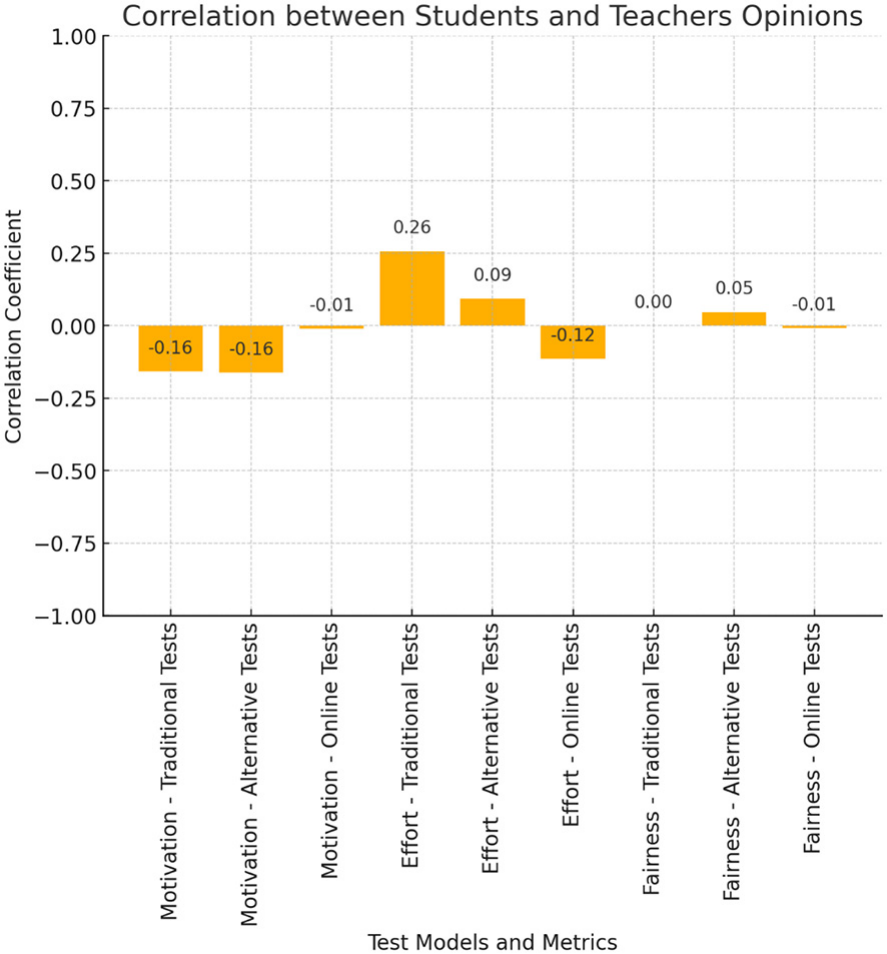
Correlation between the students' and teachers' opinions about different test models. The figure shows that student-teacher correlations in motivation scores were weakly negative for traditional (*r* = −0.158) and alternative assessments (*r* = −0.163), while effort scores were positively correlated in traditional assessments (*r* = 0.256).

To investigate the agreement between student and teacher perceptions, Pearson correlation coefficients were calculated over basic dimensions such as motivation, fairness, and effort. Most correlations are weak, indicating different perspectives across the board (Figure 4).

### Correlation matrix and intrinsic relationships

**Figure 5:**
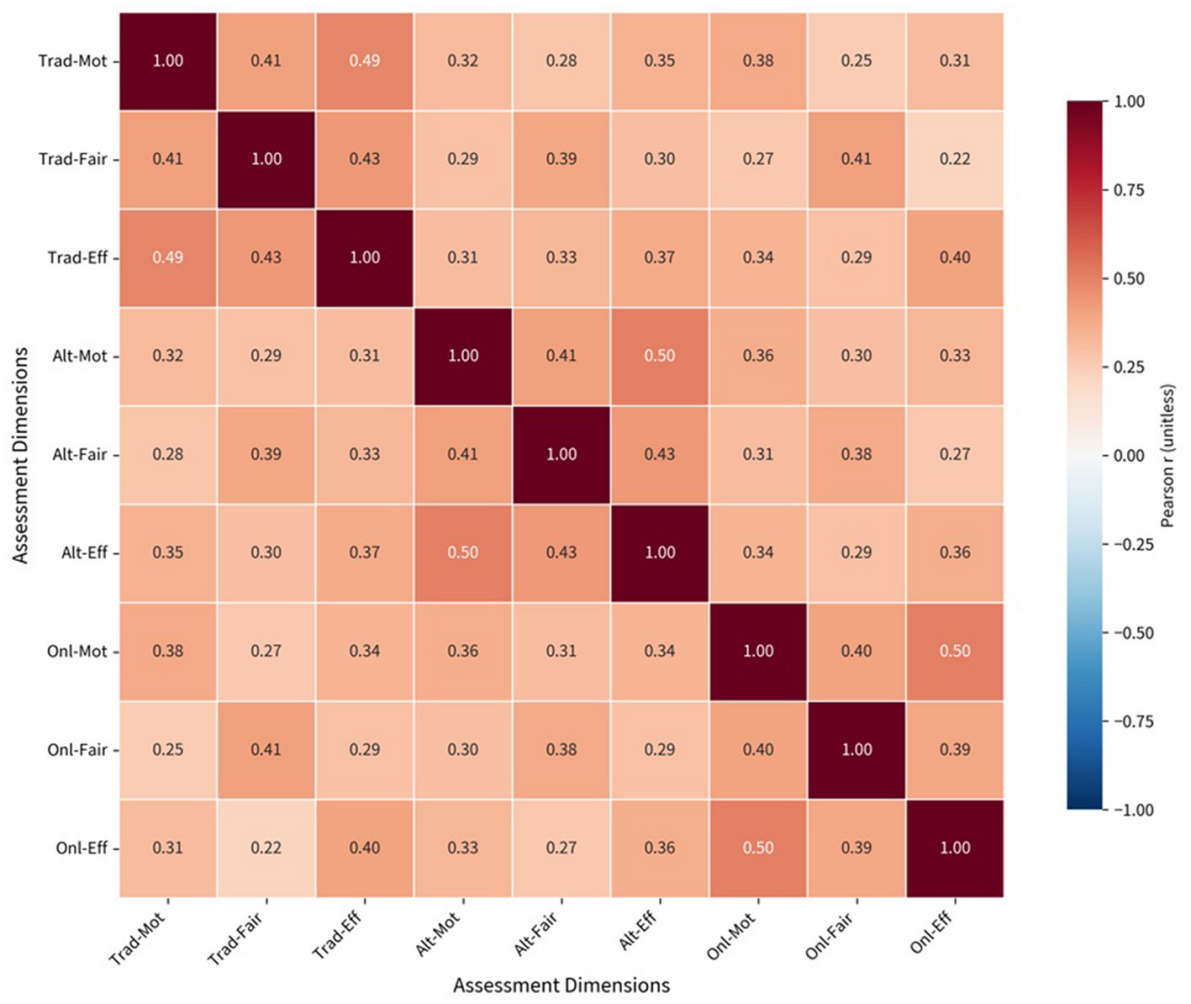
Correlation matrix heatmap on teachers' and students' attitudinal stances. The heatmap reveals strong positive correlations between motivation and effort within the same test type: alternative assessments (*r* = 0.50), online assessments (*r* = 0.50), traditional assessments (*r* = 0.49). Fairness is moderately correlated with motivation (*r* = 0.41) and effort (*r* = 0.43) for alternative assessments.

A correlation matrix was constructed to examine the internal relationships between motivation, effort, and fairness across test types (Figure 5). The heatmap revealed strong positive correlations between motivation and effort within the same test type, specifically:

- Alternative assessments (*r* = 0.50),
- Online assessments (*r* = 0.50),
- Conventional assessments (*r* = 0.49).

## Discussion

This study provides a comparative analysis of how students and teachers evaluate traditional, alternative, and online assessments in terms of motivation, fairness, and effort, contributing to the growing body of research on perceptions of language assessment. The findings highlight significant differences in stakeholder perspectives, with implications for the design and implementation of balanced assessment strategies in EFL/EMI environments. The study is also discussed by comparing it with recent developments in technology mediation evaluation over the last 5 years.

### Different views on motivation and current trends

A key finding is the discrepancy between how students and teachers perceive the motivational value of different assessment types. While students rated all three formats as moderately motivating, teachers expressed more skepticism toward the motivational effectiveness of alternative assessments. This mismatch may stem from differing notions of what motivates students: students may be intrinsically motivated by innate autonomy and creativity in alternative tasks, while teachers may prioritize extrinsic motivators, such as structure and accountability, in traditional and online formats. Teachers' perception of lower motivation toward alternative assessments may reflect their belief that structured assessments (e.g., multiple-choice tests) provide better accountability and study behavior (Guo, 2025). This interpretation is speculative, as the questionnaire did not include open-ended items probing the reasons behind motivational ratings; it is offered here as a theoretically grounded hypothesis drawing on prior research on teacher assessment beliefs.

This finding is partially consistent with the meta-analysis findings of Gavrilau et al. (2025). Gavrilau et al. found that students generally have a positive attitude toward online assessments, but this attitude varies by assessment type. In particular, it is stated that structured and gamified online assessments have a stronger effect on student motivation. In this study, students' finding all three assessment models equally motivating indicates that they can adapt to different assessment formats and are open to diversity.

Teachers' perception of lower motivation toward alternative assessments is also supported by recent research. Yanik and Kiliç (2025) note that teachers are generally positive yet cautious toward AI-assisted assessment tools, yearning for the control and predictability provided by traditional assessments. Similarly, Jia and Hew (2022) show that, in addition to the perceived benefits for teachers' adoption of online assessment tools, skepticism about their impact on student motivation also plays a role.

As Guo (2025) points out, instructors' assessment preferences are shaped by their dual commitment to learning enhancement and performance monitoring—a tension often reflected in their choice of assessment tools. This study reveals a new dimension that further deepens the teachers' dilemma: technology integration. While teachers perceive online assessments as less demanding than both traditional and alternative assessments, they also place less faith in their motivational effectiveness. This suggests that technology's streamlining of assessment processes does not automatically enhance its pedagogical value.

### Perceptions of fairness and inclusivity: post-pandemic perspectives

Both students and teachers held moderate perceptions of fairness, but students were less likely to view online assessments as equally fair. This aligns with previous research indicating that students have concerns about the integrity and transparency of digital testing environments (Alenezi, 2022; Ng et al., 2021). In contrast, alternative evaluations were consistently rated as the fairest; likely due to their ability to adapt to different learner profiles and learning styles. Teachers' perception that online assessments are fairer than students perceive them to be may stem from teachers' better understanding of the technical and pedagogical rationale behind the assessment process, while students focus on the technical challenges and insecurities they encounter. This interpretation is speculative and draws on the broader literature on stakeholder differences in assessment literacy; future qualitative research could explore the specific reasoning underlying these divergent fairness perceptions.

Ng et al.'s (2021) comparative study sheds light on the reasons why online assessments are perceived as less fair by students: technical issues, lack of oversight, and the digital divide. Similar trends were observed in this study, but teachers perceived online assessments as fairer. This difference may arise from teachers having a better understanding of the technical and pedagogical rationale behind the assessment process. At the same time, students focus on the technical challenges and insecurities they experience.

Especially in multicultural contexts, perceptions of justice become even more complex. Joo et al. (2024) examined the effectiveness of AI-driven feedback systems in multicultural settings, demonstrating that cultural differences significantly influence perceptions of technology. The majority of the sample in this study of Middle Eastern and African descent calls for a deeper examination of how cultural factors shape perceptions of online assessment. The fact that alternative evaluations were considered the fairest by both groups indicates their capacity to accommodate cultural diversity.

In the post-pandemic period, does the impact of online evaluations on the perception of justice change? Gavrilau et al. (2025) note that while students' positive attitudes toward online assessments have increased over time, this increase varies by assessment type and the quality of implementation. The observed student-teacher difference in this study indicates that both the technical infrastructure and students' digital assessment literacy need improvement to increase the perceived fairness of online assessments.

### Perceived effort and interpretation variability: a technology perspective

The study revealed significant differences in perceptions of effort required across test formats. Students have found traditional assessments to be the most demanding, likely associating them with high-stakes memorization and exam preparation. However, teachers identified alternative assessments as the most cognitively and logistically challenging, likely because of the continuous engagement and deep learning they require. This divergence may stem from differing understandings of effort: students associate effort with test preparation and memorization, while teachers may view it as equating to sustained engagement and performance on complex, open-ended tasks. Teachers, in particular, have perceived online assessments as requiring minimal effort; this is likely due to simpler management procedures and lower cognitive demands on students. Students, on the other hand, may find online exams less cumbersome due to the ease of access and flexible scheduling. These interpretations are speculative and based on the broader assessment literature; the current study did not collect qualitative data to confirm the specific reasoning behind participants' effort ratings.

These results agree with Cheng et al. (2004), who found that trainers view performance-based assessments as both pedagogically valuable and resource-intensive. However, the integration of AI and automation into appraisal processes over the past few years has the potential to shift this balance. Li (2025) highlights the potential of automated writing assessment systems to reduce teacher workload. However, Li et al. (2024) note that teachers exhibit a dual-pronged attitude toward these systems—appreciating both time savings and quality concerns.

In this study, teachers perceive online assessments as requiring the least effort, indicating the advantages of automation and data management. However, this perception raises questions about the pedagogical depth of online assessments. Kessler (2024) points out that while technologies such as ChatGPT are transforming assessment practices, teachers are questioning the pedagogical value of “lightweight” online assessments. The findings of this study support this concern, suggesting a careful balance between the efficiency and pedagogical effectiveness of online assessments.

The divergence also shows how stakeholders frame “effort” differently; students often emphasize task time, while teachers focus on the depth and quality of their contributions. This difference becomes especially important in distance learning contexts. Jia and Hew (2022) state that teachers have difficulty assessing student engagement and deep learning in online assessments, so the concept of “effort” in face-to-face interaction differs in the digital environment.

### Correlation patterns and the role of fairness: in light of new findings

The correlational analysis revealed low agreement between student and teacher perceptions, reinforcing the idea that assessment is experienced differently by each group. Importantly, strong correlations between motivation and effort emerged within each group, especially within the same type of assessment. This finding suggests that when learners perceive an assessment as motivating, they are more likely to make effort aligned with motivational theories in language learning (Dörnyei, 2001). Furthermore, fairness was moderately associated with both motivation and effort, particularly for alternative assessments. This supports the premise that fairness is not just an ethical concern but a psychological catalyst for learners' investment and persistence. Ensuring that evaluation models are perceived as fair can have subsequent benefits for both motivation and achievement (Carless, 2015; Rea-Dickins and Gardner, 2000). These interpretations are theoretically grounded but speculative, as the correlational design does not permit causal inference; future experimental or longitudinal research could test whether enhancing perceived fairness directly increases motivation and effort.

Recent research expands on these findings. Gavrilau et al. (2025) indicate that the effectiveness of online assessments correlates with students' self-regulation skills and their level of digital literacy. The motivation-effort relationship observed in this study can be further strengthened in digital environments, as students have greater responsibility for controlling their own learning processes. However, this relationship can also be fragile without adequate digital support and guidance.

The integration of AI-powered evaluation systems further complicates the relationships in the justice-motivation-effort triangle. Joo et al. (2024) point out that the fairness of AI-driven feedback systems is directly related to their transparency and explainability. Systems using “black box” algorithms may be perceived as less fair by students. The strong relationship between fairness motivation and alternative evaluations observed in this study underscores the importance of transparent, explainable evaluation processes.

### Pedagogical implications and future directions

The findings of this study offer several pedagogical implications for technology-mediated language assessment. Firstly, the differences between students' and teachers' perceptions highlight the need for a collaborative, transparent approach to assessment design. Open communication about evaluation criteria, expectations, and processes can help bridge perceptual gaps.

Secondly, students perceive online assessments as less fair, suggesting that fairness should be given particular consideration in their design. In addition to technical infrastructure, oversight mechanisms, transparent evaluation criteria, and equal access opportunities should be ensured. As Gavrilau et al. (2025) suggest, the effectiveness of online assessments heavily relies on the quality of implementation.

Third, the fact that alternative assessments were rated as the most equitable and motivating by both groups supports their further integration into EMI contexts. However, teachers' concerns about the effort required by these assessments can be addressed through professional development and resource allocation. As Li (2025) points out, AI-powered tools have the potential to reduce teacher workload, but these technologies need to be integrated to enhance their pedagogical value.

Finally, the strong relationships among motivation, effort, and fairness highlight the need to design holistic evaluation ecosystems. Instead of a dependency on a single evaluation model, hybrid approaches can be adopted that combine the strengths of different models. For example, the structural advantages of traditional assessments, the fairness and motivational potential of alternative assessments, and the accessibility of online assessments can be combined to create a more comprehensive assessment approach.

Future research may extend this study's cross-sectional design by adopting longitudinal designs to examine how perceptions of assessment change over time and how they relate to learning outcomes. In particular, how will the integration of AI-powered assessment systems transform these perceptions and relationships? The role of cultural factors in perceptions of digital assessment can be explored in greater depth. Furthermore, intervention studies to improve teachers' digital assessment literacy can improve practice in this area.

## Conclusion

This study reveals systematic divergences between student and teacher perceptions of traditional, alternative, and online assessment models in a multicultural EMI context. Three key findings merit emphasis. First, students exhibit format-flexible motivation, whereas teachers display skepticism toward alternative assessments, reflecting divergent beliefs about what drives learner engagement. Second, alternative assessments command the highest fairness ratings from both groups, while online assessments suffer a fairness deficit among students that demands targeted intervention. Third, the strong motivation-effort correlation and the moderating role of fairness underscore the need for integrative assessment design that addresses psychological as well as technical dimensions.

The theoretical contribution lies in extending TAM into language assessment by demonstrating that perceived fairness functions as a critical antecedent to both perceived usefulness and engagement intention. The methodological contribution resides in the systematic comparison of three stakeholder groups across three assessment models within a single culturally heterogeneous setting. The practical contribution involves evidence-based guidance for hybrid assessment strategies that balance accountability, autonomy, and accessibility.

Educational institutions should invest not merely in digital infrastructure but in stakeholder dialogue, transparency mechanisms, and professional development that align assessment formats with the diverse expectations of multicultural learner populations. As technology-mediated assessment continues to evolve, maintaining equity and motivation at the center of design decisions will remain essential for valid and inclusive language education.

## Data Availability

The raw data supporting the conclusions of this article will be made available by the authors, without undue reservation.
